# Oxalate of Cerium in Vomiting

**Published:** 1861-01

**Authors:** Charles Lee


*Oxalate of Cerium in Vomiting.*—This article was introduced into medical use, by Dr. Simpson, of Edinboro', about a year ago, to check the vomiting of pregnancy. But more recently it has proved useful in so much wider a field, that it promises to assume a permanent place among the mineral tonics. * * * * When I began to use it, I limited it to cases of advanced pregnancy, which had resisted all the ordinary remedies, such as creasote, hydrocyanic acid, ice, bismuth, &c. I specify *advanced* pregnancy, for in no case have I seen this troublesome symptom appear before the fourth month, without yielding to creasote, or prussic acid, or better still, minute doses of sulphuric acid and brandy. * * * But, as I have remarked, the efficacy of oxalate of cerium appears by no means confined to the relief of vomiting in pregnant women. In the vomiting that often accompanies phthisis, in pyrosis, hysterical emesis, and the various dyspeptic conditions of the stomach, especially in atonic dyspepsia, I have found the effects of this remedy no less encouraging.—*Dr. Charles Lee, in the American Journal of Medical Sciences, October*, 1860.

